# Quantification of Abdominal Fat Depots in Rats and Mice during Obesity and Weight Loss Interventions

**DOI:** 10.1371/journal.pone.0108979

**Published:** 2014-10-13

**Authors:** Bhanu Prakash KN, Venkatesh Gopalan, Swee Shean Lee, S. Sendhil Velan

**Affiliations:** Laboratory of Molecular Imaging, Singapore Bioimaging Consortium, Agency for Science, Technology, and Research, Singapore, Singapore; Stanford University School of Medicine, United States of America

## Abstract

**Background & Aims:**

Obesity is a leading healthcare issue contributing to metabolic diseases. There is a great interest in non-invasive approaches for quantitating abdominal fat in obese animals and humans. In this work, we propose an automated method to distinguish and quantify subcutaneous and visceral adipose tissues (SAT and VAT) in rodents during obesity and weight loss interventions. We have also investigated the influence of different magnetic resonance sequences and sources of variability in quantification of fat depots.

**Materials and Methods:**

High-fat diet fed rodents were utilized for investigating the changes during obesity, exercise, and calorie restriction interventions (N = 7/cohort). Imaging was performed on a 7T Bruker ClinScan scanner using fast spin echo (FSE) and Dixon imaging methods to estimate the fat depots. Finally, we quantified the SAT and VAT volumes between the L1–L5 lumbar vertebrae using the proposed automatic hybrid geodesic region-based curve evolution algorithm.

**Results:**

Significant changes in SAT and VAT volumes (p<0.01) were observed between the pre- and post-intervention measurements. The SAT and VAT were 44.22±9%, 21.06±1.35% for control, −17.33±3.07%, −15.09±1.11% for exercise, and 18.56±2.05%, −3.9±0.96% for calorie restriction cohorts, respectively. The fat quantification correlation between FSE (with and without water suppression) sequences and Dixon for SAT and VAT were 0.9709, 0.9803 and 0.9955, 0.9840 respectively. The algorithm significantly reduced the computation time from 100 sec/slice to 25 sec/slice. The pre-processing, data-derived contour placement and avoidance of strong background–image boundary improved the convergence accuracy of the proposed algorithm.

**Conclusions:**

We developed a fully automatic segmentation algorithm to quantitate SAT and VAT from abdominal images of rodents, which can support large cohort studies. We additionally identified the influence of non-algorithmic variables including cradle disturbance, animal positioning, and MR sequence on the fat quantification. There were no large variations between FSE and Dixon-based estimation of SAT and VAT.

## Introduction

Obesity is a medical condition contributing to major health problems including cardiovascular disease, insulin resistance, glucose intolerance, dyslipidemia, and type II diabetes. The fat distribution, with higher amount of abdominal adipose tissue, is associated with metabolic alterations [1]. There are two major compartments of abdominal fat: subcutaneous adipose tissue (SAT), which is present between the skin and the abdominal wall, and visceral adipose tissue (VAT) which surrounds the abdominal organs.

The link between VAT mass and insulin resistance is well-understood [2]; what is less clear is whether the VAT causes insulin resistance since a similar link has been shown between SAT mass and insulin resistance [3]. There is actually considerable variability in results regarding the relationship between insulin sensitivity and regional fat depots in humans. This could be due to technical issues related to measurement of the visceral fat depot [4] and/or variability in the relationship between the size of a fat depot and its lipolytic activity [5].

The above studies have shown that the VAT depot is more strongly associated with insulin resistance and the risk of developing type-2 diabetes than the SAT depot. To study these differences, in a pre-clinical phase, rodent models are widely used to monitor both the accumulation of fat during obesity and the mobilization of fat during an anti-obesity intervention [6], [7]. Imaging modalities like magnetic resonance spectroscopy (MRS) provides qualitative information about the composition of fat depots (visceral and subcutaneous), while computed tomography (CT) and magnetic resonance imaging (MRI) provides quantitative information of the fat volumes [8]. MRI is a non-invasive and non-ionizing modality making it more suitable for longitudinal and repeated measures of fat compartments. Additionally, it is versatile as it allows the visualization of the body organs, their shape, size, composition, and quantification, which helps in clinical diagnosis and treatment.

The first necessary step for fat quantification is the identification and segmentation of fat depots. Segmentation of abdominal MR images is a challenging task due to the lack of homogeneous intensity profile, complex shapes, poor edge definition, motion artifacts, the absence of models that fully capture the possible fat distribution in each structure and the low signal-to-noise ratio. Accurate segmentation and quantitation of SAT and VAT are critical for understanding the effect of the weight-loss interventions including exercise, diet, and drugs. Earlier segmentation work includes semi-automated or automated approaches for quantification of the fat depots in human [9, 10, 11, 12, and 13] and animal studies [6],[14].

Traditionally, fat segmentation techniques include threshold based separation of fat regions from the background tissues [15], [16], [17], local neighborhood information based threshold selection method [6], combinational methods [15], fuzzy logic [18] and active contours [19], [20]. Threshold based methods perform well for images with uniform intensities; however high field MR images show a large intensity variance which affects the selection of a suitable threshold. Adaptive [16] and local information [17] based thresholding methods yield better results than the conventional methods. A fully automated, three-stage fuzzy logic analysis was implemented in [13] to quantify total adipose tissue (TAT), SAT and VAT in morbidly obese humans. Yang, et al., [14] developed a segmental shape model and fuzzy logic based approach to assess the quantity and distribution of abdominal fat in mouse models. The algorithm efficiently handled the disappearing muscle layer issue. Lankton, et al., [21], [22] proposed a segmentation technique using the hybrid geodesic region-based [23] curve evolution that combines the benefits of both geodesic and the region based active contours by forming a geodesic energy from local regions around the curve. The resulting flow is more robust to initial curve placement and image noise. It is capable of finding significant local minima and partitioning the image under the assumption that the inside and outside points of the object can be modeled by the mean intensities of the local regions.

**Figure 1:**
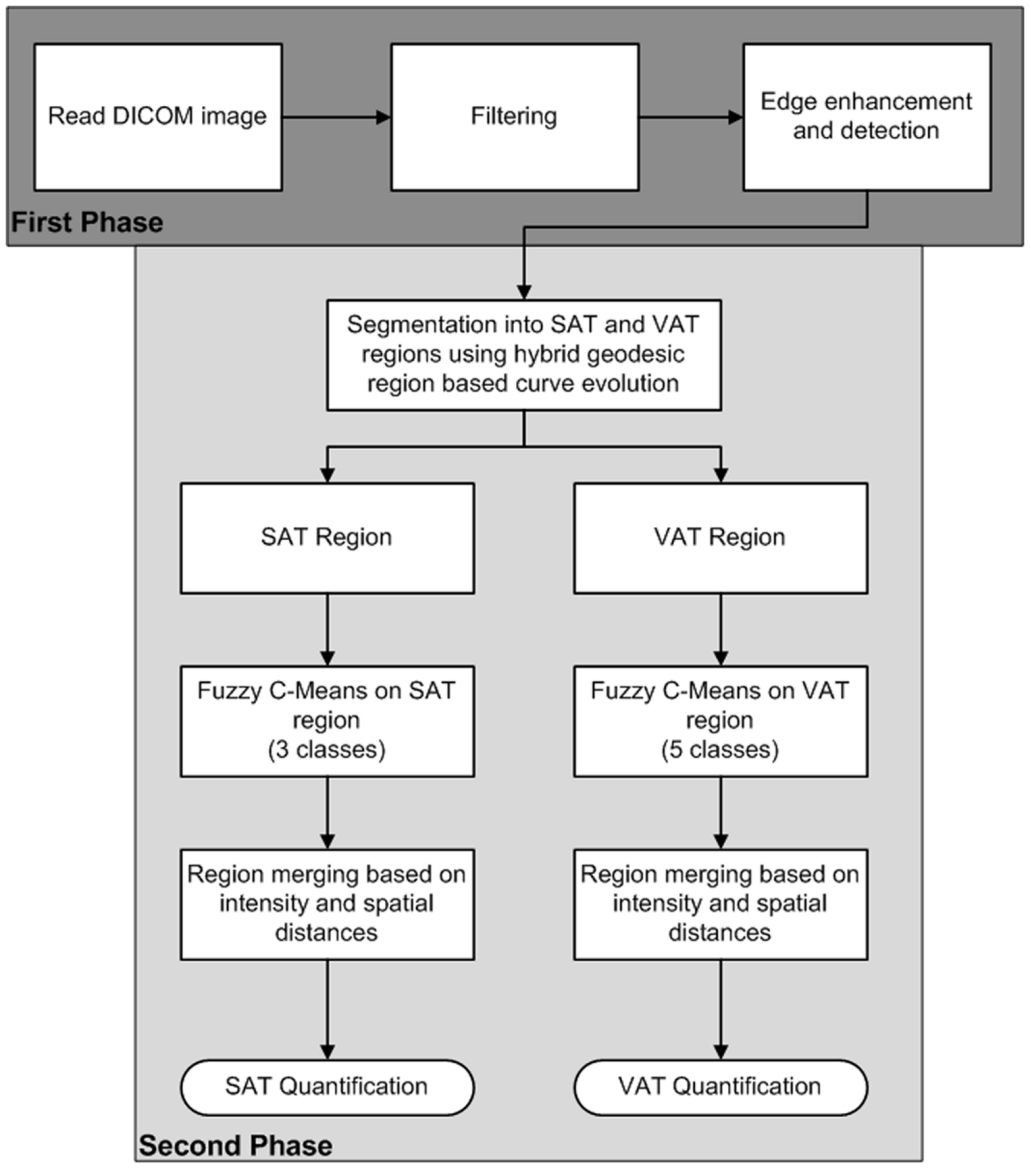
The overall flowchart describing the different stages and processing steps involved in segmentation and quantification of subcutaneous adipose tissue (SAT) and visceral adipose tissue (VAT).

The main objective of this work is to develop an automated hybrid method using modified geodesic region-based active contour and fuzzy clustering to distinguish and quantify different adipose tissues (SAT and VAT) in a large cohort of rodents during obesity and weight loss interventions. Additionally, we evaluated the influence of different MR sequences and other sources of variability on the quantification of fat depots. The flowchart describing different stages of the algorithm is shown in Figure 1. The theory and detailed explanation of geodesic curves is presented in the supplementary material Material Theory S1.

## Materials and Methods

### In Vivo Measurements

All animal experiments were approved by the institutional animal care and use committee of the biological resource center, A*STAR, Singapore. In vivo measurements were performed using a 7T Bruker ClinScan MRI/MRS scanner (IDEA VB 15). Twenty-one rats (male, F344, 5 weeks old) were split into three equal sized cohorts as a control group (CG), an exercise intervention group (EX) and a calorie restriction group (CR). All groups were fed with high–fat diet for 13 weeks.

The exercise and calorie restriction interventions were initiated when the rats were 14 weeks old, for a period of 4 weeks. In the EX group, the animals were subjected to exercise for 30 minutes, twice daily on the treadmill at 20 m/min. In the CR group, the intake of the high-fat diet was reduced by 30%. MR imaging for all three groups was performed at 14 weeks, prior to the start of the interventions, and at 18 weeks. Transverse fast spin echo (FSE) abdominal images (between L1–L5 of the lumbar vertebrae) were acquired using a volume transmit and receive coil with FOV of 65×65 mm^2^, base resolution of 320×320, TR/TE of 3573/33 ms, number of averages  = 4, intra- and inter- plane resolution of 0.2031 mm and 1.6 mm respectively. The imaging parameters were kept identical for the pre- and post-intervention scans.

In addition to the rat cohorts, abdominal images were also obtained from a cohort of (N = 7) high-fat diet fed mice. The mice (C57Bl6/J, 18 weeks) were imaged using FSE (with and without suppression) using a mouse volume transmit/receive coil, FOV of 50×38 mm^2^, base resolution of 196×256, TR/TE of 3938/42 ms, number of averages  = 2, intra- and inter- plane resolution of 0.195 mm and 1.1 mm respectively. The DIXON images were acquired with FOV of 29×37 mm^2^, base resolution of 200×256, TR/TE of 3573/33 ms, number of averages  = 2, intra- and inter- plane resolution of 0.1445 mm and 1 mm respectively, in the same position of the animal.

### Image processing and analysis

#### Problem definition

The abdominal muscular wall separates the two fat compartments - subcutaneous adipose tissue (SAT) and visceral adipose tissue (VAT) regions. Segmentation and quantification were performed in two stages; initially, separating SAT and VAT regions by a geodesic active contour, followed by, fuzzy clustering and region merging the SAT and VAT regions as shown in Figure 1. Both SAT and VAT appear hyper-intense on the FSE based MR images. The abdominal wall separating the two regions appears iso-intense and the abdominal organs appear hypo to iso-intense compared to the fat regions on FSE based MR images. The pre-processing steps included converting DICOM data into 3D image data, intensity normalization, removal of background and irrelevant features by thresholding, and 2D anisotropic diffusion filtering [24]. The edge strength was improved using edge enhancement.

#### Segmentation of SAT and VAT regions

The initial curve for the hybrid geodesic region-based curve evolution was derived from the binary image of each slice, formed by adaptive thresholding of the pre-processed image (filtered) into foreground (combined SAT and VAT regions) and background regions. The edge map of the binary image was used as the initial contour for the geodesic region-based active contour.

#### Shrinking and expansion of the contour

**Figure 2:**
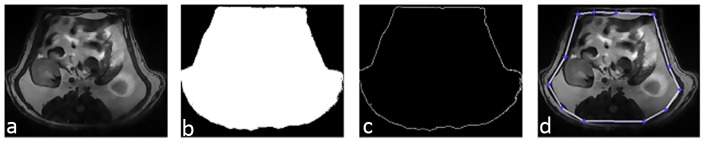
Different steps involved in deriving the initial contour for the hybrid geodesic region based curve evolution method. a) Filtered image, b) binary mask derived from the filtered image, c) edge map of the binary mask and d) placement of the initial contour after shrinking.

The initial contour of the foreground region was shrunk using a scaling factor of 0.85 to 0.75, empirically derived based on the size of the initial binary mask to adapt to the changing shapes and size of the abdominal slices, and placed it at the center of the slice. This ensured shrinkage of the hybrid contour into the abdominal area and avoided contour convergence to the background/image edge as shown in Figure 2.

The hybrid geodesic contour was allowed to expand for convergence at the boundary between SAT and VAT regions. In the expansion stage, the area of the current contour was continuously evaluated in comparison to the contour of previous iterations in order to track a steady contour expansion and its stability. If the difference was negligible or less than a predefined threshold (e.g. <10 pixels) the algorithm was terminated. If the difference was non-negligible, the algorithm was re-iterated until satisfactory results were achieved. We also implemented a hard limit on the number of iterations based on the area difference between successive iterations.

#### Fuzzy clustering and region merging

**Figure 3:**
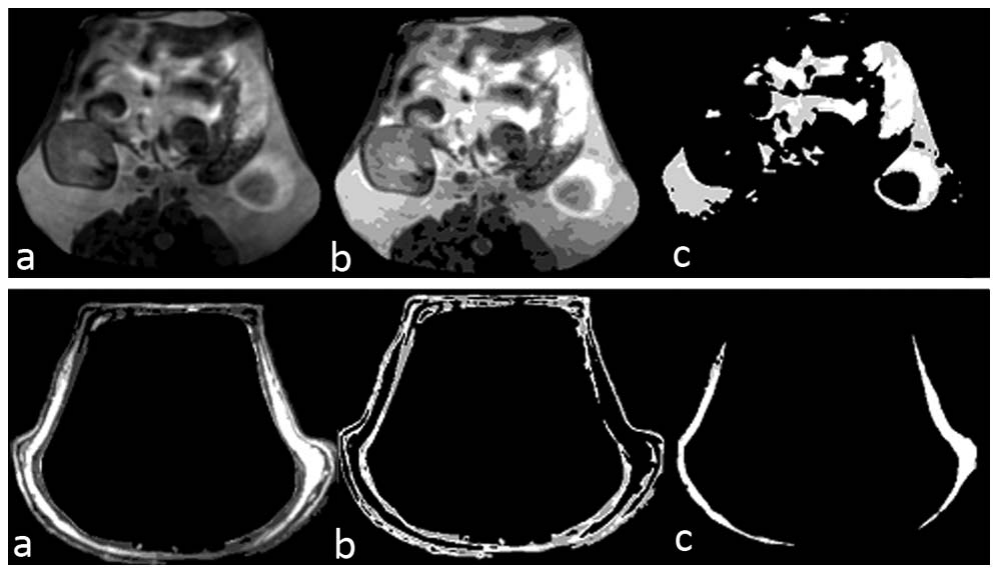
Top trace - VAT segmentation stages – a) VAT region separated by the active contour b) clustered VAT image and c) final segmentation results of VAT. Bottom trace - SAT segmentation stages – a) SAT region separated by the active contour and the clustered SAT image b) mask of the skin and the abdominal wall and c) final segmentation results of SAT.

Clustering techniques [25] are unsupervised methods that have been used to organize/classify data into groups based on the similarities of the member data items. Clustering algorithms do not rely on assumptions common to conventional statistical methods, such as the underlying statistical distribution of data, and therefore are useful in situations where minimum prior knowledge is available. As it is very difficult to exactly quantify/model the MR inhomogeneity, partial volume, noise, receiver coil sensitivity and its influence on different regions of image (inter- and intra-slice), we used fuzzy clustering (Fuzzy C- means algorithm – FCM) for grouping the SAT and VAT voxels. The number of classes for SAT and VAT was empirically selected as 3 and 5 respectively after analyzing the intensity variations in the respective regions on all the slices. The different FCM regions in SAT and VAT were merged based on the intensity and neighborhood relation (Figure 3). The voxels of skin and the abdominal wall were removed from the SAT region to get the final SAT volume (Figure 3, bottom trace). Results of segmentation were checked manually for their consistency in several data sets before applying the algorithm to the cohort study. The proposed algorithm was developed and implemented in a MATLAB 2008R environment, running on a Windows 7 environment with dual core CPU X 9650 @ 3 GHz with 2 GB RAM.

### Inter- and intra-scan variability

We performed additional scans on control rats (N = 7) to validate the segmentation and quantification algorithm for its accuracy, reproducibility, repeatability, influence of animal positioning on quantification of fat, and effects of water suppression on quantification, by implementing the following three scenarios: 1) clone scans, 2) bed – out/in scans and 3) animal change scans. In the cloned scans, the sequence was repeated without any change in parameters. In the bed – out/in scans, the images were acquired after moving the animal bed out of the bore and placing it back into the magnet without changing the animal's position. In the animal change scans, the scans were repeated after the animal was repositioned in the bed.

#### Sequence influence

Imaging using fast spin echo (with and without suppression) and Dixon was performed on obese mice (N = 7) to evaluate the fat quantification. Dixon-based fat quantification was considered as the reference for the evaluation of our results.

### Ex vivo Analysis

After the post-intervention imaging, rats were sacrificed by cardiac puncture. The various fats including subcutaneous, and gonadal, mesenteric, retroperitoneal and perirenal fat (the sum of these fat tissues was considered as visceral fat) from the CG, EX and CR groups were sampled and weighed. After computation of the SAT and VAT volumes from the abdominal images, fat mass was estimated by using the adipose tissue density that is defined as ∼0.9 g/ml [26].

### Statistical Analysis

Imaging and quantification of SAT and VAT were performed using a double-blinded evaluation. Paired sample T- test analysis was conducted to understand the significant changes in the SAT and VAT between pre-and post-intervention. The intraclass correlation analysis was performed to evaluate the consistency of the SAT and VAT quantitative measurements [27]. Comparison of fatpad and MR-based fat quantification was performed to check the correlation between the two methods. Error analysis, and correlation analysis (Pearson, Spearman and Kendall- Tau tests) were performed for both SAT and VAT quantification with clone, bed-out/in and animal change scans.

## Results

### Results of intervention

**Figure 4:**
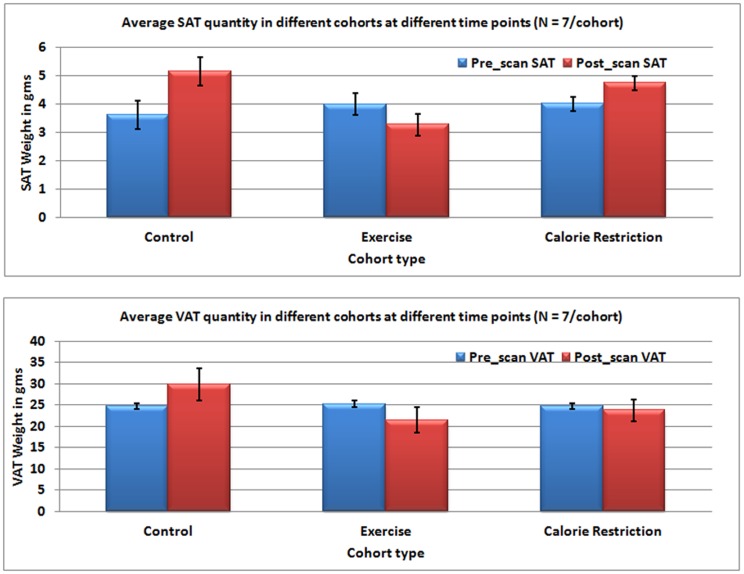
The graphs show the amount of SAT in grams for different groups (top trace) and for VAT (bottom trace) calculated using the proposed segmentation method.

**Figure 5:**
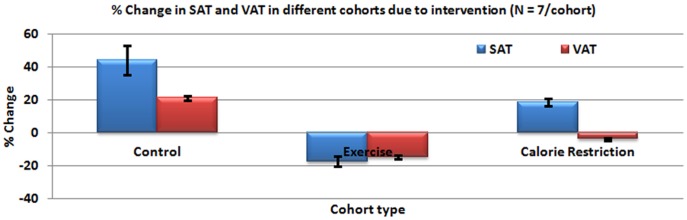
The percentage change in SAT and VAT between pre- and post-intervention scan for different groups calculated using the proposed segmentation method.

Table 1 shows the results of quantitation of fat for various cohorts. The EX and CR groups showed weight reduction (P<0.01) at 18 weeks, compared to the CG group. Figure 4 shows the pre- and post-intervention changes for SAT (top trace) and VAT (bottom trace) in grams for different groups evaluated using the proposed segmentation method. Figure 5 shows the percentage change in SAT and VAT after the intervention. The EX group showed decrease in both SAT and VAT; while CR group showed increase in SAT and decrease in the VAT.

**Table 1:** Quantification of SAT and VAT in pre-scan, post-scan and percentage of change between pre- and post-scan.

SAT			
	*Pre-scan*	*Post-scan*	*%Change*
**CG**	3.6241±0.4044	5.1591±0.4998	44.22±9.0
**EX**	3.9854±0.6158	3.2662±0.3903	−17.33±3.07
**CR**	4.0040±0.5172	4.7314±0.2475	18.56±2.05

VAT			
	*Pre-scan*	*Post-scan*	*%Change*
**CG**	24.7064±0.6810	29.8738±3.7501	21.06±1.35
**EX**	25.2776±0.6981	21.4805±2.9400	−15.09±1.11
**CR**	24.7731±0.7232	23.7721±2.5496	−3.9±0.96

Paired samples T-test between the pre- and post-scans showed significant changes in both SAT (P<0.001, 0.01 and 0.001 for control, exercise and calorie restriction cohorts respectively) and VAT (P<0.001, 0.001 and 0.05 for control, exercise and calorie restriction cohorts respectively). The intraclass correlation analysis was used to measure the consistency of SAT and VAT quantification. We observed a strong correlation and low variance in the intraclass distribution as shown in Figure S1, indicating high agreement in results of quantification for all the groups.

### Correlation of MR based SAT and VAT quantification with Fat pad Analysis

**Figure 6:**
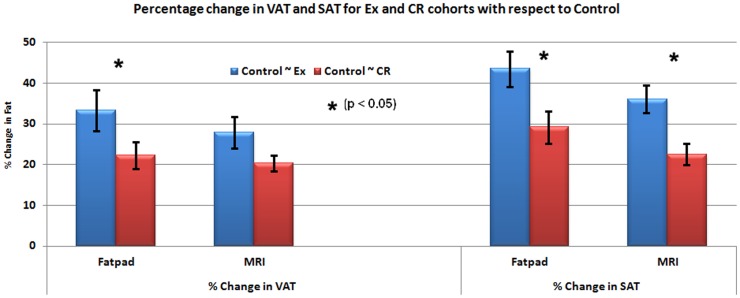
Comparitive plots of MR and fat pad based quantification of SAT and VAT for Ex and CR groups with respect to control group. The graphs indicate the percentage change in SAT and VAT in Ex and CR groups by both the methods.

Additionally to validate the results of MR based SAT and VAT quantification, we performed a correlation analysis with respect to tissue based fat pad analysis. The results of analysis are shown in Figure 6. We observed an overestimation of fat by fat pad analysis. Both MR and fat pad analysis showed similar trend and changes in fat percentage for Ex and CR groups with respect to control group.

### Inter- and intra-scan variability analysis

**Figure 7:**
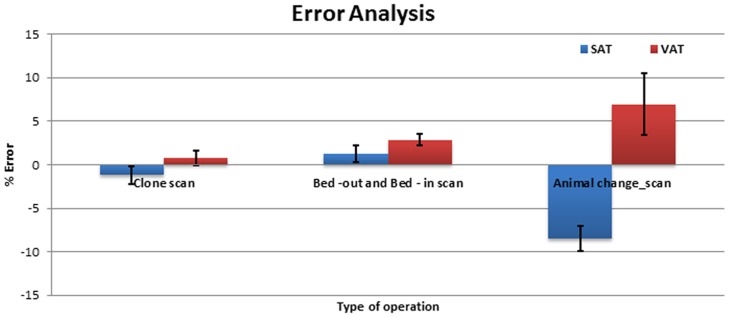
Changes in SAT and VAT in clone, bed-out and bed-in and animal change scans.

The analysis of SAT and VAT quantification with clone, bed-out/in and animal change scans are shown in Figure 7. The error was least in the clone scans and largest with animal repositioning. This larger margin of error was expected since animal placement and positioning in the bed strongly influences the imaging outcomes.

#### MR Sequence influence analysis

**Figure 8:**
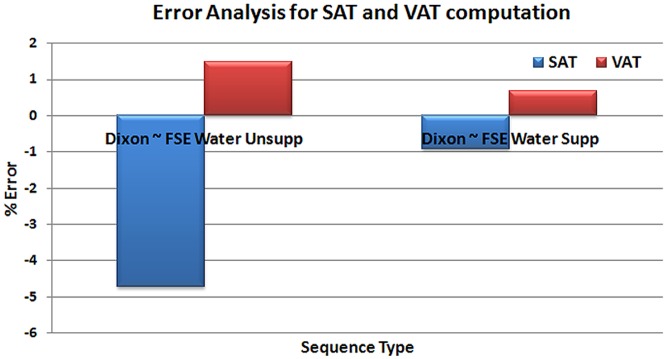
Error analysis comparing the results of quantification of SAT and VAT based on FSE (with and without water suppression) and DIXON sequences.

We evaluated FSE based imaging with and without water suppression and Dixon techniques for quantification of SAT and VAT. Results of Error and correlation analysis (Pearson, Spearman, and Kendall- Tau tests) for both SAT and VAT are shown in Figure 8, and Table 2. A strong correlation was observed between FSE based imaging and DIXON with respect to quantification of SAT and VAT. The average difference was about 1.2% for SAT and 3% for VAT between unsuppressed water based FSE and DIXON. The correlation coefficients were calculated with respect to Dixon imaging based quantification. FSE with and without water suppression had correlation coefficients of 0.9709, 0.9803 and 0.9955, 0.9840 respectively.

**Table 2:** Correlation analysis of SAT, VAT quantification using FSE (with and without water suppression), and DIXON imaging sequences.

	SAT		VAT	
Variable Y	FSE_Water_ Supp	FSE_Water_ Unsupp	FSE_Water_ Supp	FSE_Water_ Unsupp
Variable X	Dixon	Dixon	Dixon	Dixon
**Sample size**	7	7	7	7
**Correlation coefficient r**	0.9709	0.9803	0.9955	0.9840
**Significance level**	P = 0.0003	P = 0.0001	P<0.0001	P = 0.0001
**95% Confidence interval for r**	0.8103 to 0.9958	0.8681 to 0.9972	0.9688 to 0.9994	0.8920 to 0.9977
**Spearman's coeff of rank correlation (rho)**	0.964	0.964	1.000	1.000
**Significance level**	P = 0.0005	P = 0.0005	P<0.0001	P<0.0001
**95% Confidence Interval for rho**	0.771 to 0.995	0.771 to 0.995	1.000 to 1.000	1.000 to 1.000
**Kendall's Tau**	0.905	0.905	1.000	1.000
**Significance level**	P = 0.0069	P = 0.0069	P = 0.0027	P = 0.0027
**95% Confidence Interval for Tau**	0.556 to 1.000	0.556 to 1.000	1.000 to 1.000	1.000 to 1.000

## Discussion

Quantity of SAT and VAT in the abdomen are important metabolic measures as they are correlated to insulin resistance and blood chemistry. In this study, we developed an automatic method to distinguish and quantify different adipose tissues (SAT and VAT) of abdomen in a large cohort of rodents to understand the influence of exercise and calorie restriction on distinct adipose tissue compartments in rats rendered obese by a high-fat diet. Additionally, we studied the influence of different MR imaging sequences and operational variability on the reproducibility and accuracy of our automatic segmentation algorithm in quantification of fat.

### Accuracy of segmentation

Our segmentation algorithm benefits from the advantages of both geodesic and region-based active contour techniques [21], [22]. Our approach thus permits correct solutions using the weak assumptions about global image properties. With our addition of pre-processing and modifications to the original algorithm (using the image features from local regions), our method is now more robust to noise; it automates the generation and placement of the initial curve and reduces the convergence time of the initial curve to the abdominal wall.

The consistency and accuracy of the segmentation was more than 80%. The coefficient of variation (COV) was [0.11, 0.15], [0.14, 0.1], [0.12, 0.12] for SAT quantification in pre- and post-intervention for the CG, EX and CR groups respectively. The VAT quantitation had similar results with values of COV [0.05, 0.08], [0.08, 0.03], [0.1, 0.06] for pre- and post-intervention. The values of COV and intra-class correlation emphasize that the quantitation by the proposed segmentation algorithm is more reproducible.

Despite its advantages, the performance of geodesic energy based segmentation is strongly dependent on the initial curve placement. While the proposed algorithm reduces the above dependence, it is still necessary to initialize the contour nearby the object to be segmented or risk that the final segmentation results will converge at an incorrect local minima. There are certain cases where, due to skin folding or abdominal compression, the abdominal wall between the SAT and VAT is not characterized by a separation of image intensities. The algorithm may therefore fail to localize the abdominal wall and might converge to a local optimum. The size of the neighborhood can also have a significant impact on the results. For our study, all of these parameters were empirically tuned to work optimally for SAT and VAT separation by checking against different images used in the study.

### SAT and VAT changes

We observed a reduction in body weight in rats that underwent exercise and calorie restriction compared to the control group. As expected, the increase in VAT and SAT was highest in the CG group fed with a high-fat diet and not subjected to any anti-obesity intervention. The calorie restriction group showed an increase in SAT (18%) and decrease in VAT (−3.9%), with the SAT gain unevenly distributed with larger deposits in the L1-L2 region. Conversely, the exercise group showed decrease in both SAT (−17%) and VAT (−15%). Interestingly, we observed an opposite distribution of fats from the previous scenario, with a higher reduction of SAT and VAT in the lower abdominal region. These findings are in line with existing literature that exercise and calorie restriction can modulate the fat composition in VAT and SAT differently [28]–[30]. Human studies [30], [31] have shown that subjects with exercise intervention for 3–4 days a week compared to 1–2 days lost more subcutaneous fat.

In addition, we have compared the MRI based quantification of SAT and VAT in L1–L5 with the fat-pad analysis. Similar trend was observed in both fat-pad and MRI based analysis for the percentage change of fat in EX and CR cohorts when compared with the CG. We found overestimation of SAT and VAT by fat-pad analysis when compared to MRI based quantification (Figure 6). Though fat-pad analysis is more useful for estimating fat in different organs it is very difficult to accurately extract the SAT and VAT from L1–L5 regions due to the mobility of fat. This might lead to contributions from the neighboring regions in turn resulting in overestimation of fat.

### Variability Analysis

Different MR sequences and operational variability influence the reproducibility and accuracy of any segmentation algorithm. Results of image segmentation and quantification also depend on the quality of image acquisition. Amongst other variables, drift in magnetic field, noise, errors in cradle positioning, animal respiration, body movements, and animal positioning may influence the quality of image acquisition.

The variability in quantification, as expected, was minimal in the cloned MR sequence where the animal position is not disturbed. We observed about 1% variation in SAT and about 2.8% in VAT quantification with the bed -out/in procedure, where the histogram of the volume data showed a shift in intensity values of the voxels. This could be due to variation in the adjustment of magnetic field homogeneity with room temperature shims. Larger errors in both SAT and VAT values were observed when the animal was removed and re-positioned in the cradle. These errors may be due to change in position/angle of the animal, magnetic field homogeneity and other physiological noise characteristics.

### Quantification based on FSE and Dixon

We observed a high correlation in the SAT and VAT quantification based on FSE-based imaging and Dixon-based sequences as shown in Table 2. The SAT quantification based on FSE with and without water suppression was higher by about 1% and 4% respectively when compared to the Dixon-based quantification. This was due to inclusion of the abdominal wall region along with the SAT and may be due to elevated intensity levels of the voxels (due to partial volume) near the SAT region. On the other hand, the VAT quantification using FSE with and without water suppression underestimated by about 0.8% and 1.5% respectively when compared to the Dixon-based method. This was due to exclusion of low intensity voxels near the abdominal organs. Though overall agreement in segmentation is high between the FSE and Dixon MRI sequences, the choice of sequence can influence the quantification of SAT and VAT due to changes in the MR properties. The FSE imaging techniques cannot purely separate the fat tissues as compared to Dixon imaging. In all cases, the success of segmentation depends on the quality of the initially acquired images.

The water and fat have a strong dependence on spin-lattice relaxation T_1_, spin-spin relaxation T_2_, and diffusion characteristics. Depending on the imaging sequence and field strength, image acquisition parameters can influence the image contrast between water and fat. Image contrast can also be affected by magnetization transfer effects, *J*-modulation effects, production of stimulated echoes and direct saturation effects, and due to differential attenuation of spatial frequencies. Multi-echo based MRI sequences for producing T_2_-weighted images have been utilized for fat quantitation [32]. The use of multiple refocusing pulses generates subtle effects (ghosting) that are not seen in conventional single echo based imaging. Water images will necessarily be T_2_ weighted whereas fat images will have an intensity, which depends on factors including J modulation. Chemical shift saturation techniques (e.g. water suppression) can also have drawbacks due to magnetic field and RF field inhomogeneity, and thus are suboptimal when imaging over large field of view, off-isocenter locations, or near interfaces between soft tissue and body cavities. The Dixon approaches do not suffer from these chemical shift saturation drawbacks, as they provide uniform separation of fat and water. It has indeed been shown that the multi-point Dixon approaches can result in a more robust separation of water and fat even with strong B_0_ inhomogeneity [33].

### Conclusions

We have developed an automated segmentation algorithm for estimation of SAT and VAT in rodents including both rats and mice. Our automated image analysis and segmentation technique will be very valuable for the analysis of large cohorts with different obesity and anti-obesity interventions due to greatly (4x) reduced image analysis time, improved accuracy, and elimination of operator variability errors. We have furthermore shown that the choice of MRI sequence, animal positioning and cradle disturbance influence fat quantification. These quantification errors are minimized when image acquisition is free from artifacts, noise, and magnetic drift. Finally, the difference between the FSE (with and without water suppression) and Dixon based quantification did not show large variation for estimation of VAT and SAT in rodents.

## Supporting Information

Figure S1 **The intra-class distribution of SAT and VAT during pre- and post-intervention scan for different groups calculated by the proposed segmentation method.**
(TIF)

Material Theory S1 **Theory of geodesic region based curve evolution.**
(DOCX)
