# Supplementary figures and images for: Quantification of Abdominal Fat Depots in Rats and Mice during Obesity and Weight Loss Interventions
Source: PLoS One. 2014 Oct 13;9(10):e108979. doi: 10.1371/journal.pone.0108979 (PMC4195648; doi:10.1371/journal.pone.0108979)

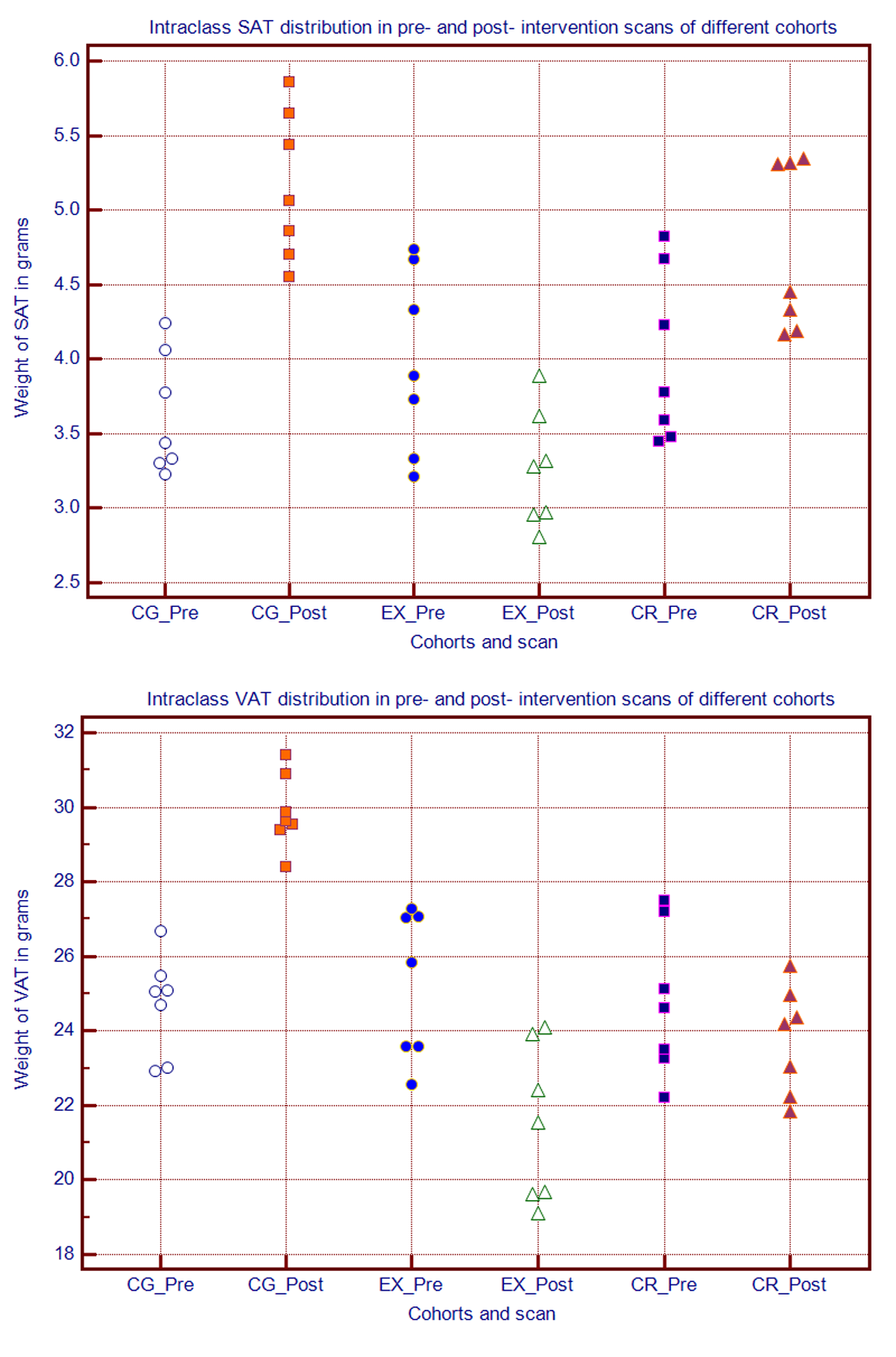

Supplement: Figure S1 — The intra-class distribution of SAT and VAT during pre- and post-intervention scan for different groups calculated by the proposed segmentation method. (TIF) [file pone.0108979.s001.tif]
