# Supplementary material for: Quantification of Abdominal Fat Depots in Rats and Mice during Obesity and Weight Loss Interventions
Source: PLoS One. 2014 Oct 13;9(10):e108979. doi: 10.1371/journal.pone.0108979 (PMC4195648; doi:10.1371/journal.pone.0108979)
Supplement: Material Theory S1 — Theory of geodesic region based curve evolution. (DOCX) [file pone.0108979.s002.docx]

**Supplementary Material**

**Theory of geodesic region based curve evolution**

Active contour models, colloquially known as snakes, are energy-minimizing curves that deform to fit image features. Snakes converge to the local minima of the image’s potential energy [19]. The internal constraints of the snake model produce tension and stiffness, which keep the model smooth and continuous, and prevent the formation of sharp corners. The external constraints influence the converging process, which can be derived using the image information or by user interaction. In some cases, an additional pressure term is added to make the models expand like balloons. Active contour models are classified as either parametric or geometric based on their representation and implementation. The parametric active contours are represented explicitly as parameterized curves in a Lagrangian framework, while the geometric active contours are represented implicitly as level set functions that evolve in an Eulerian framework.

The classical approach of active contours is based on deforming an initial contour by function minimization in order to increase or decrease the energy thus moving the curve toward a local maxima or minima (i.e. towards the boundary of the object to be detected). These active contours are examples of the general technique of matching deformable models to image data by means of energy minimization. The energy function is composed of two components, one controls the smoothness of the curve, and another attracts the curve towards the boundary. In the recent models of active contours based on the theory of curve evolution and geometric flows, the initial curve propagates with a velocity using the regularization and expansion/shrinking functions. Such models are defined by a geometric flow based on mean curvature motion. It allows automatic changes in the topology when implemented using the level-sets based numerical algorithm [20].

Geodesic active contour reformulates the standard active contour problem into finding local minimum cost curves on a Euclidean metric using the intrinsic geometric properties based curve parameterization. This results in dependence of the energy on the length and smoothness of the curve without the need of an additional regularizing term. Whereas in region based active contours approach, the contour energies are defined using the regional properties with the assumption that when a curve is defined/placed on the object border the image is partitioned into two or more distinctive regions.

The hybrid energy proposed by Shawn Lankton et al [21, 22] combines the benefits of both geodesic and the region based active contours by forming a geodesic energy from local regions around the curve. The resulting flow is more robust to initial curve placement and image noise, but also capable of finding significant local minima and partitioning the image. The key assumption made in the formulation is at each point on the object edge the nearby points inside and outside of the object can be modeled by the mean intensities of the local regions.

A geodesic active contour takes the standard active contour form and reformulates the problem into finding local minimum cost curves on a Euclidean metric.

***Energy Definition and Curve flow***

The definition for the energy of the curve ***E*** is given by the following function

(1)

where ***C*** represents the evolving curve, and ***I*** represents the image data. Here, ***f*** is any positive, decreasing function of the image data. Its values form the metric over which the minimum length geodesic will be found as the curve deforms. One of the standard selection for ***f*** is the spatial gradient ($\nabla\hat{I}$ ) of a Gaussian smoothed image ***I*** as shown below.

(2)

The hybrid energy equation with geodesic energy from local regions around the curve is defined as follows.

(3)

where Ω and $\overline{Ω}$ represent the region on the interior and exterior of the curve respectively, and are the arithmetic means of the local neighborhoods, function *X* evaluates to 1 in a local neighborhood defined by a small radius and 0 elsewhere, and ***s*** parameterizes the curve and specifies every point along the curve as the contour integral is evaluated. The curve deformation uses a gradient descent for energy minimization using the variational calculus to compute the time derivative of hybrid energy equation [21, 22 ].
